# Ultrasound-Guided Percutaneous Intercostal Cryoneurolysis for Acute-on-Chronic Pain in CLOVES Syndrome

**DOI:** 10.7759/cureus.34066

**Published:** 2023-01-22

**Authors:** Sudipta Sen, Johanna B De Haan, Mariam Mehrafza, Nadia Hernandez

**Affiliations:** 1 Anesthesiology and Perioperative Medicine, McGovern Medical School University of Texas Health Science Center, Houston, USA; 2 Anesthesiology, McGovern Medical School University of Texas Health Science Center, Houston, USA

**Keywords:** cloves syndrome, chronic pain, acute pain, intercostal nerves, cryoneurolysis, regional nerve blocks, chronic and acute pain management

## Abstract

Cryoneurolysis is an analgesic method that has been shown to provide extended pain relief in postoperative patients. However, to date, this method has not been described in nonsurgical inpatients with chronic pain experiencing an acute exacerbation. This analgesic modality has the potential to provide pain relief for patients whose expected duration of severe acute pain would outlast that of other regional anesthetic techniques while avoiding opioid escalation and facilitating discharge.

We present a patient with acute exacerbation of chronic pain from breast ulcerations caused by congenital lipomatous overgrowth, vascular malformations, epidermal nevis, spinal/skeletal anomalies/scoliosis (CLOVES) syndrome that was successfully treated as an inpatient with a portable cryoneurolysis device.

This is the first reported use of cryoneurolysis in an inpatient setting to treat acute-on-chronic pain in a nonsurgical patient. The authors recommend regional anesthesiologists and acute pain specialists to utilize this technique to provide analgesia in patients with complex pain to facilitate hospital throughput.

## Introduction

Congenital lipomatous overgrowth, vascular malformations, epidermal nevis, spinal/skeletal anomalies/scoliosis (CLOVES) syndrome was initially described as a spectrum of overgrowth syndromes requiring multiple extensive debulking and reconstructive surgeries with morbid postoperative sequelae [1]. Clinical diagnosis of CLOVES syndrome is confirmed by genetic analysis of the mosaic-activating mutation in gene PIK3CA [2]. Patients with CLOVES syndrome have a poor quality of life due to recurrent severe infections from lymphatic malformations, and they are at high risk for rapid progression of lesions requiring surgery and multiple hospitalizations [3].

Pain management is an integral part of treatment for patients with CLOVES disease. CLOVES patients have multiple interventions to control the lipomatous overgrowth and vascular malformations using embolization, sclerotherapy, rapamycin chemotherapy, surgical excision, reconstruction, and skin grafting [4]. Thus, CLOVES patients are likely to develop tolerance and dependence on opioids and/or develop chronic pain. Managing acute exacerbations of chronic pain is challenging and requires a multidisciplinary approach that includes pharmacological therapy, behavioral modifications, and interventional procedures [5,6].

Cryoneurolysis is an effective method for providing extended relief of acute postoperative pain in patients undergoing thoracotomy [7]. A new handheld device for cryoneurolysis has made this technology portable and feasible to use in a hospital setting. Ultrasound guidance for percutaneous cryoneurolysis makes this procedure similar to an ultrasound-guided nerve block [8]. We report a case of a patient with chronic pain from breast ulcerations caused by CLOVES syndrome presenting with severe acute pain that was successfully treated with ultrasound-guided percutaneous intercostal cryoneurolysis.

This case report was previously presented at the 46th Annual Regional Anesthesiology & Acute Pain Medicine meeting in May 2021, and informed consent was obtained for publication from the patient.

## Case presentation

**Figure 1:**
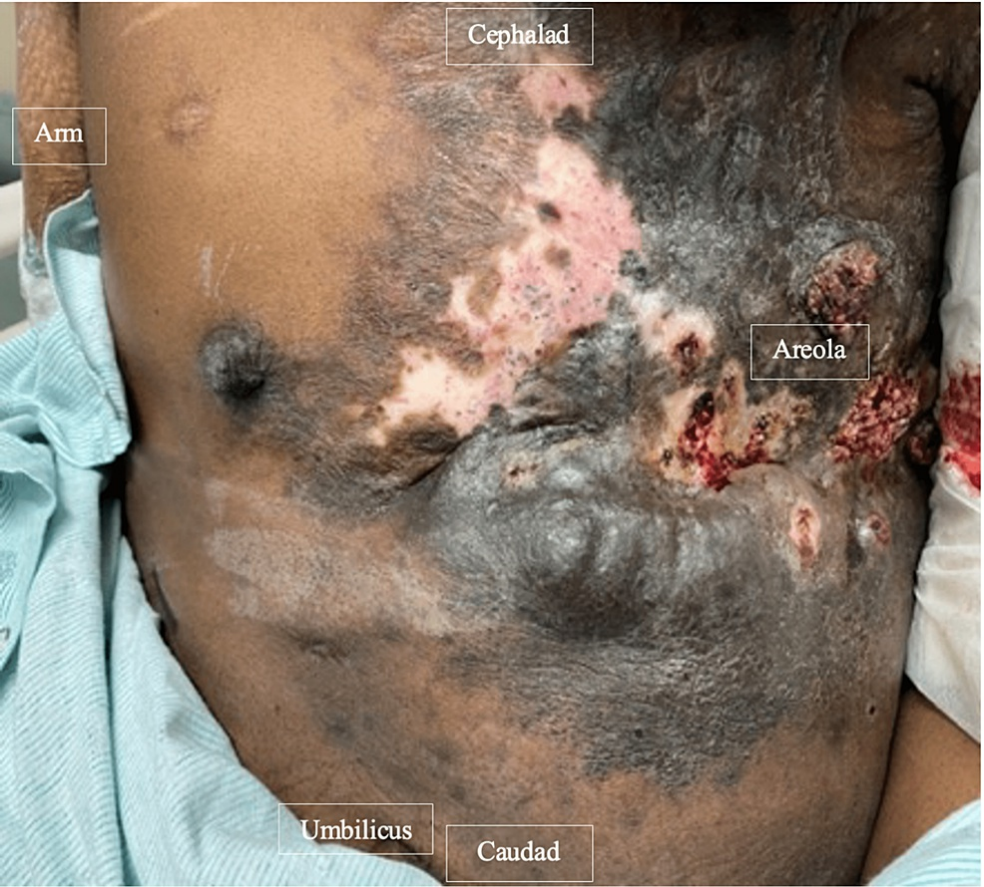
Bilateral anterolateral chest wall illustrating ulcerations and previous scarring secondary to suspected CLOVES syndrome CLOVES: Congenital lipomatous overgrowth, vascular malformations, epidermal nevis, spinal/skeletal anomalies/scoliosis.

A 30-year-old female with a history of diabetes mellitus, anxiety, depression, lymphangioma of the left axilla, and chronic pain due to bilateral anterolateral chest wall ulcerations secondary to suspected CLOVES syndrome presented to the emergency room with severe acute bilateral anterior chest wall pain. She had previously undergone multiple surgeries for tumor debulking including bilateral mastectomy, chest wall reconstruction, debridement, and skin grafting to the thoracic wall. Upon evaluation, she attributed her acute-on-chronic pain to recurrent cellulitis and acute ulceration of the anterior and lateral chest walls (Figure 1). The patient also presented with severe nausea, vomiting, and decreased oral intake. In the setting of severe, intractable pain, the patient was initially managed with an evolving multimodal pain regimen that included escalating opioid doses with no significant improvement in pain symptoms (Table 1).

**Table 1:** Opioid consumption and verbal pain scores during hospitalization * indicates cryoneurolysis. Prior to cryoneurolysis, days 0, 1, 2, and 3 had average numeric pain scores and opioid consumption in MME. Cryoneurolysis was performed on day 4, and the patient was discharged from the hospital on day 5. MME: Morphine milligram equivalents.

Hospitalization Day	Opioid Consumption (MME)	Average Verbal Pain Score (x out of 10)
Day 0	48	10/10
Day 1	46	10/10
Day 2	72	10/10
Day 3	78.4	10/10
Day 4*	56*	6/10*
Day 5	0	0/10

On hospital day 2, the primary team implemented patient-controlled analgesia (PCA) with hydromorphone to manage her pain. Despite a total opioid consumption of 72 morphine milligram equivalents (MME) over the initial 24 hours, there was no pain relief. At this time, our acute pain service was consulted for assistance with pain management with the goal of discharging the patient on a safe and lasting pain regimen that would allow time to establish care with an outpatient chronic pain specialist. Upon initial assessment, she described her pain (10 out of 10 on the numeric pain scale) as constant, unrelenting, and excruciating, localized to the bilateral chest wall and abdomen with neuropathic symptoms. She initially declined regional anesthesia, so oral methadone was added to her multimodal regimen. Despite adjustment of the multimodal pain regimen, the patient reported no improvement in pain over the next 48 hours. On hospital day 4, she agreed to interventional pain management, and we performed ultrasound-guided percutaneous cryoneurolysis of the left eighth through tenth and right fourth through sixth intercostal nerves corresponding to the areas of the patient’s chest wall pain. 

**Figure 2:**
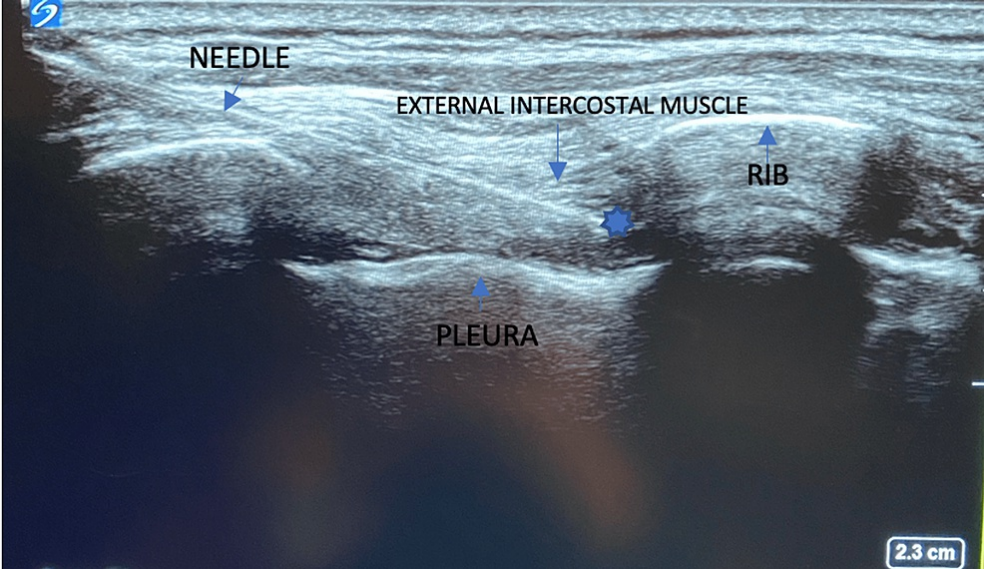
Ultrasound-guided image of thoracic 4 intercostal cryoneurolysis

For the procedure, the patient was placed in a lateral decubitus position. The arm was adjusted to protract the scapula to improve visualization of high thoracic intercostal spaces. A high-frequency linear transducer was placed on the patient’s back in a sagittal orientation to obtain a short-axis view of the corresponding ribs and intercostal space. The patient was given intermittent boluses of versed, fentanyl, and ketamine to tolerate the procedure. The overlying skin was prepped with chlorhexidine and anesthetized with 1% lidocaine. Bilateral ultrasound-guided single-shot paravertebral blocks (PVB) were performed with 20 milliliters of 0.25% bupivacaine hydrochloride to provide analgesia for the cryoneurolysis. For the cryoneurolysis, an 18-gauge, 48-mm angiocath was advanced in-plane under ultrasound guidance toward the internal intercostal muscle under the rib. The needle was retracted leaving the angiocath within the muscle. The 20-gauge, 90-mm Smart Tip 190 (Iovera Smart Tip, Pacira Biosciences, FL, USA) was introduced through the angiocath to approach the right T4 intercostal nerve (Figure 2) as the initial target site. Two cycles of 106 seconds of freezing were applied, and the formation of the ice ball was visualized under ultrasound. This procedure was then repeated at the right T5, T6, and left T8, T9, and T10 intercostal levels.

Following the cryoneurolysis procedure, the patient felt an overall improvement in symptoms. After 24 hours post-procedure, once the PVB had worn off, the patient reported a pain score of 0/10 and required no opioid analgesics. In addition, the patient had an improved affect and mood that correlated with the significant analgesia she experienced. She reported that the right-sided chest pain had completely resolved, while the left side was no longer characterized by a sharp pain. The patient was discharged with a prescription of tramadol as needed for breakthrough pain and a follow-up with an outpatient chronic pain specialist. Despite the significant relief of pain symptoms expressed following the cryoneurolysis procedure, the patient did not follow up with a chronic pain physician and returned to the emergency department four weeks later with the return of severe chest wall pain.

## Discussion

Procedures performed by regional and acute pain specialists are relatively short in duration, usually lasting long enough to provide analgesia for severe acute postoperative pain. The options currently available to provide extended thoracic wall analgesia for outpatients are indwelling fascial plane catheters or single-shot blocks with liposomal bupivacaine, both of which only last a few days [9]. This is the first report of the successful use of cryoneurolysis to treat acute-on-chronic pain in an inpatient setting unrelated to surgery in which analgesia was provided for several weeks. We chose cryoneurolysis for this patient because the expected duration of her severe acute pain would have outlasted the pain relief provided by liposomal bupivacaine or the ambulatory elastomeric pump reservoir of 400-700 mL used with indwelling catheters available at our institution. In addition, socioeconomic issues made it challenging to schedule an immediate follow-up with a chronic pain specialist.

Cryoneurolysis is a procedure that involves the direct application of cold temperature (-20°C to -100°C) to cause reversible interruption to nerve axons with the goal of achieving a prolonged analgesic effect as the nerves regenerate over weeks to months. Wallerian degeneration or axonotmesis occurs distal to the lesion with inhibition of afferent and efferent signal transmission [10]. The endoneurium and myelin sheath are left intact, thus classified as a Sunderland type 2 injury [11].

Cryoneurolysis was historically accomplished using fluoroscopy and surgical dissection. The new, handheld, percutaneous cryoprobe uses nitrous oxide as a cryogen. Liquid nitrous oxide flows into the tip of the cryoprobe and expands into a gas causing a drop in temperature and the formation of an “ice ball” at the target site. One cycle of freezing is adequate for small nerves as ultrasound guidance allows visualization of nerves and improves the precision of cryoprobe placement. The use of ultrasound guidance is particularly helpful as partial cryoneurolysis of a nerve can result in exacerbation of symptoms if it does not cause sufficient axonotmesis [12].

Freeze-thaw cycles have been shown to increase the size of the ice ball if the nerve cannot be visualized well on ultrasound or is large in diameter. The handheld cryoprobe can achieve a temperature of up to -88°C. The duration of cryoanalgesia varies and depends on the amount of axonal disruption achieved. Axonal disruption is determined by the length of the cycle, the temperature achieved, and the actual distance of the ice ball from the terminal branches of the peripheral nerve.

In our patient, we marked areas of intense pain on the bilateral anterior chest wall and targeted the corresponding intercostal nerves for intervention. The success of the procedure was evidenced by the patient’s dramatic drop in numeric pain scores, opioid consumption prior to discharge, and readiness for discharge after the intervention. Upon return to the emergency room, the patient reported analgesia for approximately four weeks following targeted intercostal cryoneurolysis. However, there have been studies that suggest a longer duration of analgesia in some patients. Green et al. studied the effectiveness of percutaneous cryoanalgesia on 43 patients with post-herpetic neuralgia or intercostal neuralgia, utilizing a single four-minute freeze cycle at -60°C and found that 50% of the patients experienced significant pain relief for a duration of three months [13].

Any regional anesthesia and acute pain procedure can result in complications. Cryoneurolysis can cause hypoesthesia, muscle weakness, and decreased proprioception in the distribution of the affected nerve. Neuritis, neuroma formation, and pneumothorax have also been reported. Given the state of our patient’s overall psychosocial status, we determined that the benefits of the procedure greatly outweighed the potential risks. Specific contraindications for this intervention include concomitant Raynaud’s disease, cold urticaria, paroxysmal cold hemoglobinuria, or cryofibrinogenemia due to the low temperatures used to freeze nerves. Standard guidelines for superficial peripheral regional anesthesia in the patient receiving antithrombotic therapy must be followed [14]. So far, one case of myonecrosis and abscess formation following percutaneous cryoneurolysis is reported in the literature [15].

## Conclusions

In this article, we report the successful use of a portable, percutaneous cryoneurolysis device in a hospital setting for the treatment of acute-on-chronic pain to facilitate opioid weaning and hospital discharge in a nonsurgical patient. More studies are warranted to establish the feasibility, safety, cost-effectiveness, and efficacy of the use of cryoneurolysis in an inpatient setting. Regional anesthesiologists and acute pain specialists who possess the skills to perform ultrasound-guided interventions can use cryoneurolysis as a potential tool for the management of complex pain to facilitate hospital discharge in opioid-tolerant patients admitted primarily for pain management.
